# Ultrasensitive tau biosensor cells detect no seeding in Alzheimer’s disease CSF

**DOI:** 10.1186/s40478-021-01185-8

**Published:** 2021-05-26

**Authors:** Brian D. Hitt, Jaime Vaquer-Alicea, Victor A. Manon, Joshua D. Beaver, Omar M. Kashmer, Jan N. Garcia, Marc I. Diamond

**Affiliations:** 1 Center for Alzheimer’s and Neurodegenerative Diseases, Peter O’Donnell Jr. Brain Institute, UT Southwestern Medical Center, Dallas, TX USA; 2 Department of Neurology, UT Southwestern Medical Center, Dallas, TX USA

**Keywords:** Alzheimer’s disease, Tau, Seeding, Cerebrospinal fluid, Biomarkers

## Abstract

Tau protein forms self-replicating assemblies (seeds) that may underlie progression of pathology in Alzheimer’s disease (AD) and related tauopathies. Seeding in recombinant protein preparations and brain homogenates has been quantified with “biosensor” cell lines that express tau with a disease-associated mutation (P301S) fused to complementary fluorescent proteins. Quantification of induced aggregation in cells that score positive by fluorescence resonance energy transfer (FRET) is accomplished by cell imaging or flow cytometry. Several groups have reported seeding activity in antemortem cerebrospinal fluid (CSF) using various methods, but these findings are not yet widely replicated. To address this question, we created two improved FRET-based biosensor cell lines based on tau expression, termed version 2 low (v2L) and version 2 high (v2H). We determined that v2H cells are ~ 100-fold more sensitive to AD-derived tau seeds than our original lines, and coupled with immunoprecipitation reliably detect seeding from samples containing as little as 100 attomoles of recombinant tau fibrils or ~ 32 pg of total protein from AD brain homogenate. We tested antemortem CSF from 11 subjects with a clinical diagnosis of AD, 9 confirmed by validated CSF biomarkers. We used immunoprecipitation coupled with seed detection in v2H cells and detected no tau seeding in any sample. Thus we cannot confirm prior reports of tau seeding activity in the CSF of AD patients. This next generation of ultra-sensitive tau biosensors may nonetheless be useful to the research community to quantify tau pathology as sensitively and specifically as possible.

**Supplementary Information:**

The online version contains supplementary material available at 10.1186/s40478-021-01185-8.

## Introduction

Intracellular aggregates of the microtubule-associated protein tau define Alzheimer’s disease (AD) and related neurodegenerative tauopathies. In AD, tau progressively accumulates in defined patterns that involve brain networks [6]. This has been hypothesized to occur via formation of aggregate “seeds” in a single neuron or group of neurons that exit and gain entry to neighboring or synaptically connected cells. The seeds then serve as templates for amplification of specific pathological tau assemblies [17, 18]. Our lab developed the first generation of tau “biosensor” cells to detect pathological seeds, here termed “v1.” These express the tau repeat domain (RD: aa244-368) containing a single disease-associated mutation (P301S) fused to cyan and yellow fluorescent proteins (CFP/YFP) [21]. Exposure of biosensor cells to pathological forms of tau (monomer or larger assemblies) induces Tau RD-CFP/YFP aggregation that creates fluorescence resonance energy transfer (FRET) [13, 21]. This may be detected by microscopy or flow cytometry [15, 21]. Biosensor cells spontaneously take up free tau aggregates, or “naked” seeds [20], but admixture with a cationic lipid-based transfection reagent such as Lipofectamine 2000 increases seeding by ~ 100–1000-fold [21]. We have previously used the original biosensor cells to detect tau seeding activity in mouse and human brain prior to neurofibrillary tangle pathology. This indicates that seed formation is perhaps the earliest detectable tau-related pathological event [16, 24], and thus could be an excellent disease biomarker. Reliable detection of seeding activity in peripheral fluids such as CSF or blood from living subjects has not yet been established, but could improve specific diagnosis of tauopathy.

Numerous studies have sought to amplify tau seeds from cerebrospinal fluid, either using biosensor cells [37], in vivo propagation in a mouse model [36], or in vitro amplification assays such as real-time quaking-induced conversion (RT-QuIC) [30, 31]. Three groups have reported detection of seeding activity in antemortem CSF of living individuals [31, 36, 37], but the sensitivity and specificity of these approaches are not established. Most mass spectrometry (MS) studies have observed that tau in the CSF contains the N-terminal and/or mid-region of tau, but not the RD [2, 8, 33]. Tau RD forms the core of tau oligomers and fibrils and is required for seed formation [9, 11]. However a recent study reports that AD CSF contains higher concentrations of tau regions within the RD than previously measured, and that levels of a fragment at the C-terminus of RD correlate well with disease progression [23]. In this study we have created a highly sensitive second generation of biosensors and tested their ability to detect tau seeding in antemortem human AD CSF.

## Materials and methods

### Generation of biosensor cell lines (Tau RD(P301S)v2L and Tau RD(P301S)v2H)

We used the previously described lentiviral FM5-YFP plasmid containing the tau segment 246 to 378 with the P301S mutation [32], replaced the human ubiquitin C (Ubc) promoter with a human cytomegalovirus (CMV) promoter, and replaced the YFP sequence with a cerulean 3 or mClover3 coding sequences. The sequence linking the tau segment and the coding sequence of the fluorophore (Cer or Clo) was optimized to the following sequence: GSAGSAAGSGEF [40]. Low passage HEK293T cells (P5) were thawed and passaged with antibiotic free media twice before co-administration of lentivirus encoding tau RD(P301S)-Clo/Cer. After four passages, single cells were cell sorted by FACS based on low or high signal for both mCerulean3 and mClover3, termed version 2 low (v2L) or version 2 high (v2H). Monoclonal colonies were expanded and characterized as described. Second generation biosensors were used according to established protocols [21].

### Western Blot

We prepared cell lysates by resuspending frozen cell pellets (~ 1 million cells) in 100µL of 0.25% Triton X-100 with protease inhibitors and incubating for 30 min on ice, followed by centrifugation at 21,000×*g* for 15 min. We adjusted clarified supernatants to a concentration of 1 mg/mL as determined by Pierce 660 nm assay and SDS-PAGE was performed with 5 µg of total protein loaded onto a 4–20% BisTris gel. After transferring the protein to a PVDF membrane, we blocked it with 5% milk in 0.1% TBS-T for 1 hr at room temperature. To detect tau protein we used HJ10.3 (David Holtzman, Washington University, St. Louis, MO), a mouse monoclonal antibody that binds the RD of tau (amino acids 250–268), at a 1:10,000 dilution in blocking buffer for 4hrs at RT. To detect the fluorescent proteins fused to tau, we used the Rockland anti-GFP antibody (cat. 600-101-215), which binds all GFP variants used in this work, at a 1:10,000 dilution, in blocking buffer. After blotting with appropriate secondary antibodies and imaging, we stripped and reblotted the membranes for GAPDH with (6C5, Fisher cat.NC9537307) at a 1:5000 dilution in blocking buffer.

### Recombinant tau fibrils

We synthesized wild-type full-length (2N4R) tau and purified it as previously described [14]. We incubated 8 µM purified recombinant tau with 8 µM heparin and 10 mM DTT at 37C for 48 h in 10 mM HEPES, 100 mM NaCl, PH 7.4. We verified the quality of fibrils by transmission electron microscopy.

### Human AD and mouse brain tissue

All mice were housed and cared for according to the UT Southwestern animal care and use guidelines and all applicable U.S. laws governing animal research. Mice: Wild type C57BL/6 (stock #00,064, Jackson Laboratory), Tau knockout (stock #007,251), and PS19 mice expressing human 1N4R tau with the P301S mutation under control of the mouse prion promoter (Prnp) [43] (stock #008,169) mice, all 9 months old. We transcardially perfused all mice, then removed and immediately flash-froze the brains in N_2_(l). We obtained frozen frontal cortex human brain tissue from 5 cases with a histopathological diagnosis of AD from the brain bank of the Alzheimer’s Disease Center UT Southwestern. We homogenized brain tissue in 10% w/vol of 1 × TBS with protease inhibitor cocktail (Roche) at 4 °C using a dounce homogenizer followed by intermittent probe sonication (Omni International) for 10 min. We centrifuged homogenates at 21,000×*g* for 15 min at 4 °C to remove cellular debris and determined protein concentrations by BCA assay (Thermo Fisher).

### Human CSF

We obtained human lumbar CSF from the UT Southwestern O’Donnell Brain Institute Biorepository, along with clinical data including age, sex, and CSF t-tau, p-tau, and Aβ_42_ levels as measured by ADmark clinical assay (Athena Diagnostics).

### Immunoprecipitation from CSF

50 μl of Dynabeads Protein A (Thermo Fisher) were washed per the manufacturer protocol and incubated with 10 μg of polyclonal antibody (TauA, Diamond Lab, available upon request) targeting the first microtubule-binding repeat of tau for 1 h at room temperature. We then added washed beads to 1 or 5 ml of human CSF and incubated them with rotation overnight at 4 °C. We eluted captured proteins in low pH elution buffer (Pierce) and neutralized the buffer with 1:10 1 M Tris pH 8.5 with a final volume of 120 μl.

### Seeding assays

Cells were passaged every 48-72 h and never allowed to reach > 80% confluence. We found that over-confluence and sparse passage conditions both reduced cell health and contributed to background false positive FRET and variability in FRET measurements. This was particularly true of the v2H cells. We plated v1 P301S [21] or v2L or v2H HEK biosensor cells in 96-well plates at 20,000 cells per well 24 h before treatment. We allowed dilutions of recombinant tau fibrils or brain homogenates in Opti-MEM (Thermo Fisher), 30 μl total volume, or immunoprecipitation eluents 120 μl total volume, to come to room temperature. We mixed 1.5 μl of Lipofectamine 2000 transfection reagent (Invitrogen) with 28.5 μl of Opti-MEM for each sample and incubated it at room temperature for 5 min before mixing it with the sample. After incubating the mixtures at room temperature for 30 min, we divided them among 3 wells of a 96-well plate. After 48 h, we trypsinized and fixed cells in 2% PFA and suspended them in flow cytometry buffer (1 × HBSS, 1% FBS, 1 mM EDTA). We determined percent FRET positivity of each well by flow cytometry as previously described [15].

## Results

### Construction of tau RD(P301S) v2H biosensor cells

**Fig. 1:**
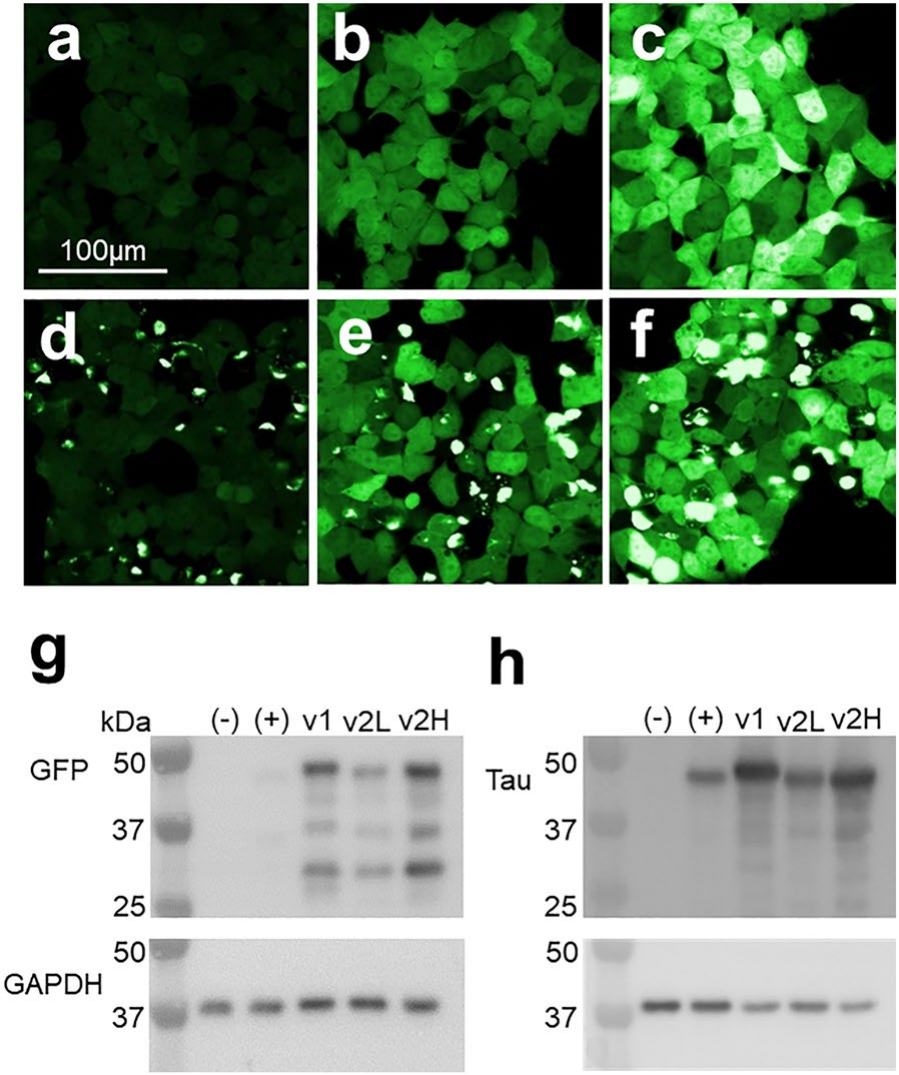
v2H biosensors express more tau-fusion protein. Fluorescent micrographs of P301S v1 (**a**, **d**), v2L (**b**, **e**), and v2H (**c**, **f**) cells demonstrate an increase in fluorescence of both v2 cell lines over first-generation biosensor cells, and of v2H over v2L when examined under the YFP channel. **d**–**f** show cells treated with synthetic tau fibrils delivered by Lipofectamine 2000. Western blot against tau (**g**) and GFP (**h**) demonstrates higher levels of tau-fusion protein in v2H biosensor cells compared to v2L cells. Negative control (−) represents lysate from naïve HEK293 cells and positive control ( +) represents lysate HEK293 cells with a single incorporation of the tau RD-mClover construct. The slightly higher molecular weight of the tau-YFP fusion in v1 cells is due to a longer linking sequence in the original fusion protein

Upon sequencing of the plasmid used to express tau RD (P301S)-CFP/YFP in the original biosensor line, we found an error in the Kozak sequence that could reduce translation efficiency. In addition to correcting the sequence, we replaced the human ubiquitin (hUBC) promoter with the cytomegalovirus (CMV) promoter, and exchanged CFP and YFP for brighter variants mClover3 (Clo) and mCerulean3 (Cer) [1, 28]. We cloned the new construct into a lentiviral expression vector, selected single colonies for characterization, and picked two clones with low and high tau expression respectively, both having minimal background FRET and with strong aggregation in response to exogenous seeds. The version 2 low-expressing clone (v2L) reliably produces FRET upon exposure to tau seeds with a dose–response curve that is highly linear with higher concentrations of tau seeds (Additional file 1: Fig. 1). The version 2 high-expressing clone (v2H) expresses higher levels of tau, and is more sensitive, with a highly linear dose response at very low concentrations of tau seeds. The v2L and v2H tau biosensors each express higher levels of intact tau RD-Clo/Cer fusions than the original biosensor line, as detected by Western blots against tau-RD and GFP, and by fluorescence microscopy (Fig. 1). (Note: Upon publication, the cells will be deposited at ATCC as Tau RD(P301S)v2L and Tau RD(P301S)v2H biosensors.)

### Increased biosensor sensitivity

**Fig. 2:**
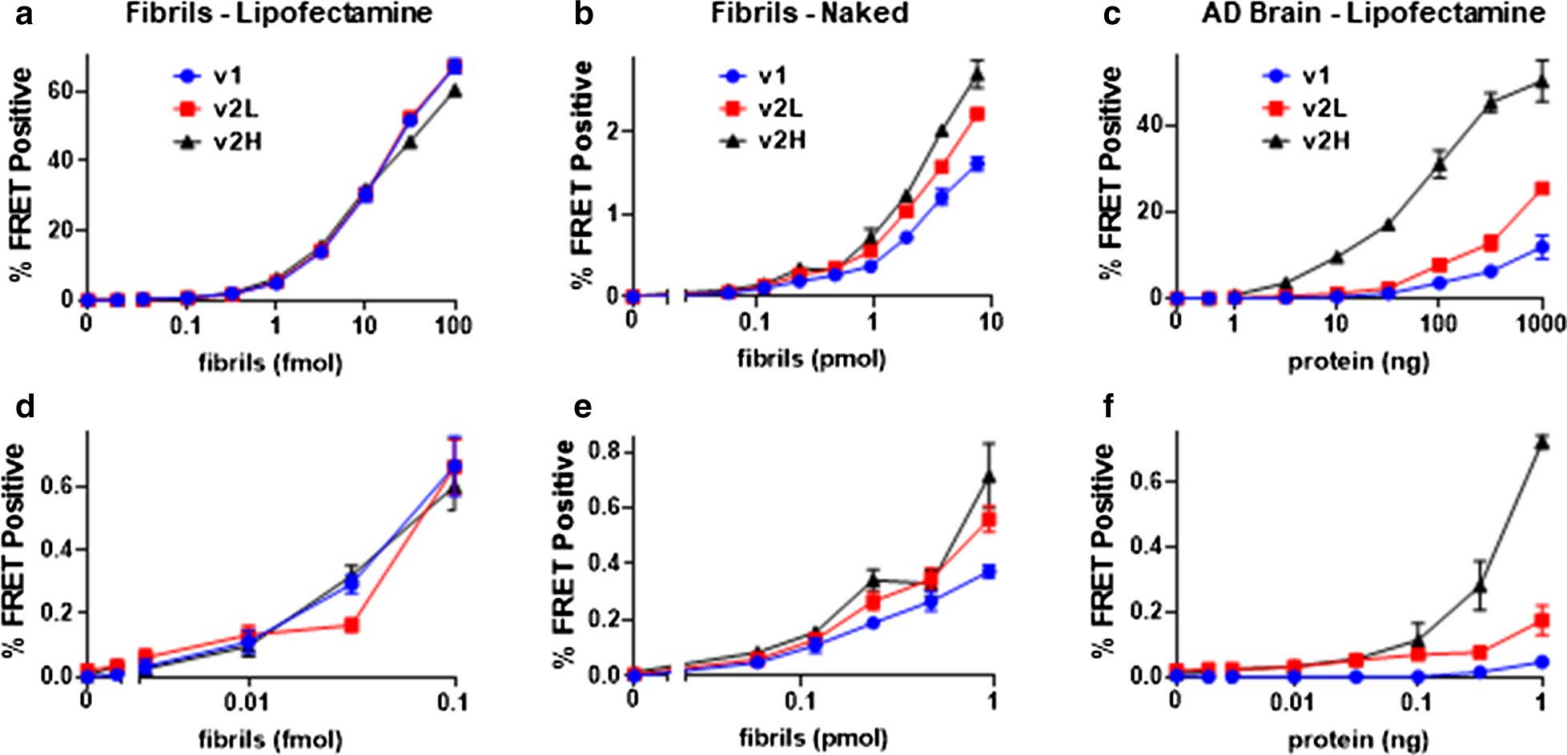
P301S v2H cells exhibit increased sensitivity to AD-derived tau seeds. The FRET assay was performed with successive dilutions of synthetic tau fibrils mixed with Lipofectamine 2000 (**a**) and without transfection reagent (**b**) (log scale), and of immunopurified tau from an AD brain with Lipofectamine 2000 (**c**). **d**–**f**) are expansions of the dose response curves of in (**a**–**c**), respectively. Lower limit of detection (LLD—defined as the lowest quantity of tau fibrils that produces a signal of FRET positivity statistically distinguishable from background, T-test, *p* < 0.05) with Lipofectamine-mediated synthetic fibril seeding was 10 amoles for all cell lines. For naked synthetic fibril seeding the LLD was 0.59 pmol for all cell lines, and with AD brain protein it was 1 ng for v1, 100 pg for v2L, and 10 pg for v2H (represent pre-IP quantities of total protein). Error bars are SEM of 3 technical replicates over which each sample was divided

We first compared the sensitivity of v2L and v2H cells to the original line (v1). We created synthetic fibrils based on exposure of recombinant tau protein to heparin [20]. We also immunopurified tau from an AD brain homogenate (AD1) using the TauA polyclonal antibody, which was raised against the first repeat of the wild-type human tau protein. We incubated fibrils with cells in the absence or presence of Lipofectamine 2000, and AD tau in the presence of Lipofectamine 2000 for 48 h, and quantified the percentage of FRET-positive cells using flow cytometry as per standard protocol [15] to determine the lower limit of detection. We express lower limits of detection in terms of absolute quantities of seed source material (synthetic tau fibrils or tauopathy brain protein) rather than concentration, since use of the assay with biological samples involves immunopurification of tau which can be performed from a wide range of volumes, and may involve tau assemblies of a range of sizes. We detected 10 attomoles monomer equivalent and 59 femtomoles monomer equivalent of synthetic tau fibrils in all cell lines with (Fig. 2a, d) and without (Fig. 2b, e) transfection reagent, respectively. In contrast, with AD-derived tau, we detected seeding from 1 ng total protein equivalent with v1 cells, but 100 pg and 10 pg with v2L and v2H cells, respectively (Fig. 2c, f), representing an increased sensitivity of tenfold for v2L and 100-fold for v2H over the original line [21]. Further, v2L and v2H had complementary ranges over which FRET response to total AD protein was highly linear (Additional file 1: Fig. 1). Kinetics were optimally linear in v2L cells between 316 pg and 100 ng (r^2^ = 0.997), and in v2H cells between 316 fg and 316 pg (r^2^ = 0.998). Given its lower limit of detection of AD seeds and improved signal to noise and linearity of kinetics in the low range, cell line v2H was used for subsequent experiments.

### Detection of brain-derived tau seeds

**Fig. 3:**
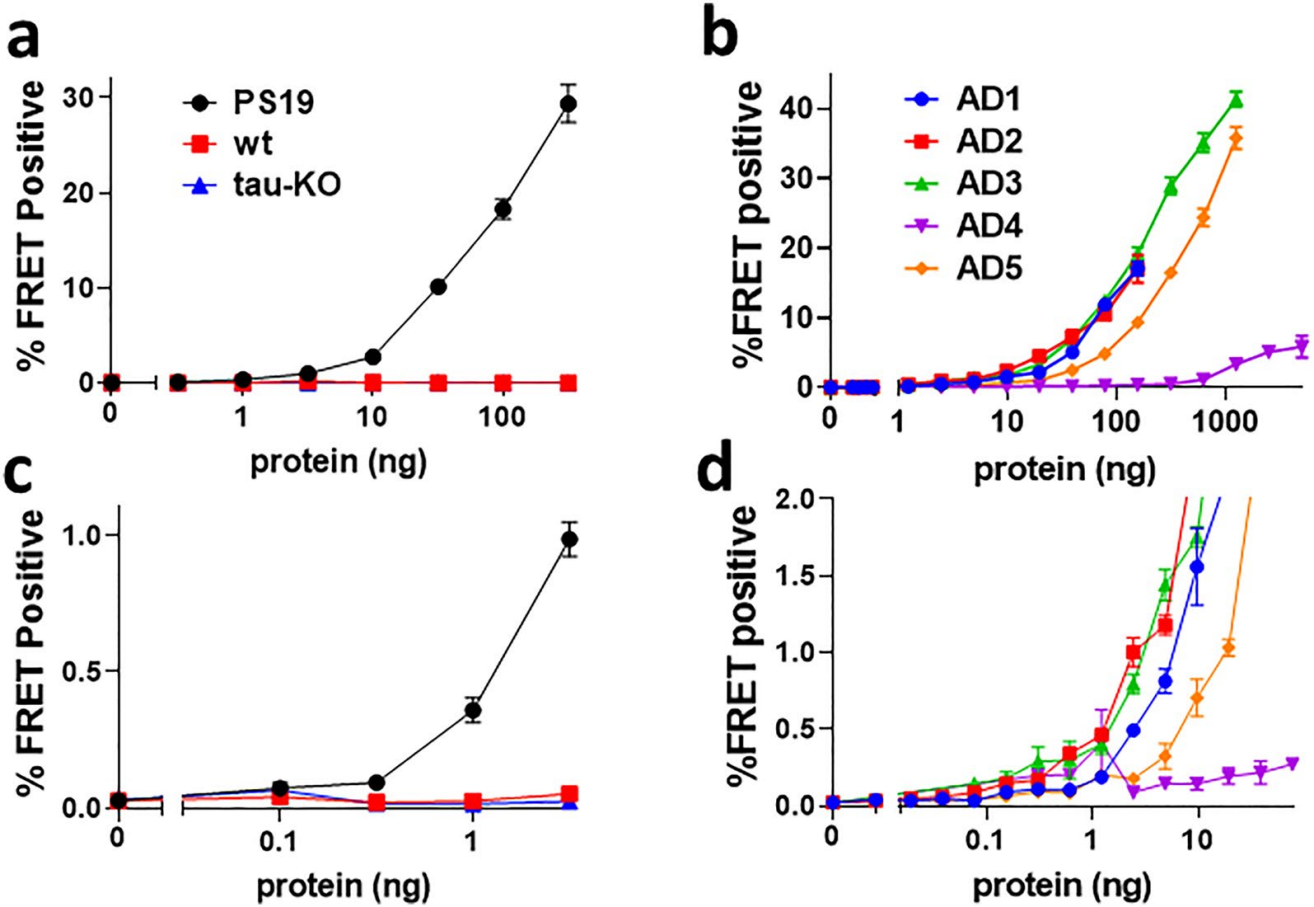
High sensitivity detection of tau seeding from biological sources. **a** Dose response curves of P301S v2H cells with protein from PS19 transgenic, wild-type, and tau knock-out mouse brain. **b** Dose response curves using protein from frontal lobe tissue of 5 AD cases. **c, d** Expansions of (**a**, **b**), respectively. LLD was 316 pg for PS19 brain, 305 pg for AD1, 153 pg for AD2, 1.2 ng for AD3, 78 ng for AD4, and 1.2 ng for AD5. Error bars are the SEM of 3 technical replicates over which each sample was divided

To evaluate detection of mouse brain-derived tau seeds, we serially diluted brain extract (10%w/v) from a 9 month-old PS19 transgenic mouse, which expresses full-length (1N4R) human tau containing the P301S mutation [43]. We transduced v2H cells using Lipofectamine 2000 (Fig. 3a, c), and measured seeding activity over four log orders of concentration. The lower limit of detection for lysate was ~ 316 pg of total protein. Next we evaluated frontal cortex homogenates from 5 AD cases. The lower limit of detection ranged from 153 pg to 1.2 ng of total protein (Fig. 3b, d).

### Efficient purification and detection of tau seeds from CSF

**Fig. 4:**
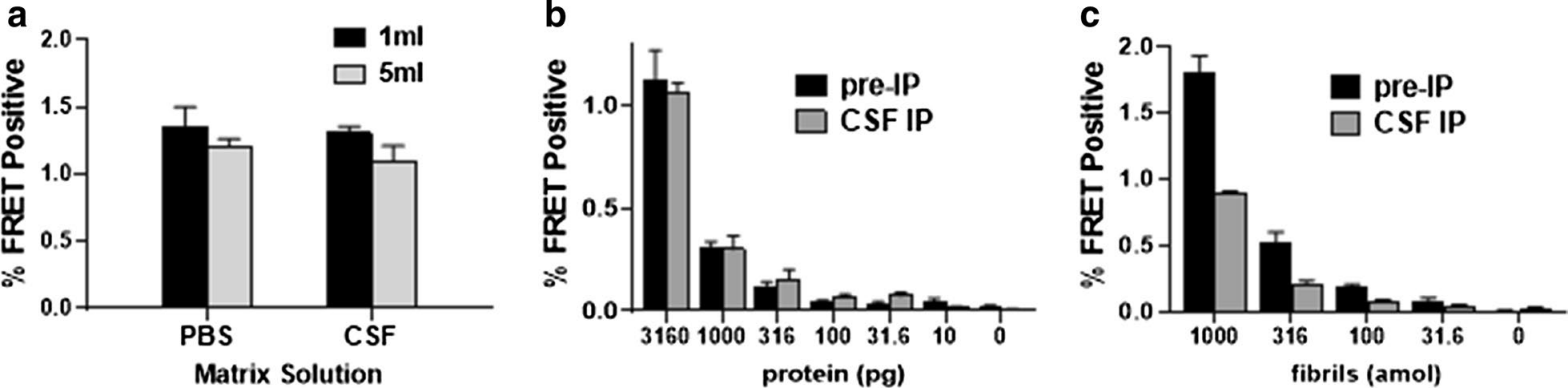
Efficient purification of tau seeds from CSF. **a** 10 ng of protein from frontal cortex of case AD1 was spiked into control CSF or PBS. FRET positivity resulting from IP followed by seeding assay of spiked samples did not differ between CSF and PBS, or with volume of IP. **b** 1 ml aliquots of control CSF were spiked with a serial dilution of protein from brain AD1. **c** 1 ml aliquots of control CSF were spiked with a serial dilution of recombinant tau fibrils. Seeding was detected from these spiked samples down to 31.6 pg of total AD brain protein and 100 attomoles of recombinant fibrils (monomer equivalent). Pre-IP shows FRET positivity from direct treatment with the same amount of protein spiked into the corresponding sample. Error bars are SEM of 3 technical replicates over which each sample was divided

To determine the lower limit of detection of tau seeds in CSF, we spiked control CSF with small quantities of AD frontal cortex protein (brain AD1) or recombinant tau fibrils. We have tried in numerous ways to seed biosensor cells directly with raw CSF, but in all cases this has led to significant toxicity and death of biosensor cells and has failed to produce seeding. Direct treatment also limits the volume of CSF that can be tested. We therefore concentrated and purified the AD seeds using a rabbit polyclonal antibody directed against tau RD (TauA), which we have found to be the most efficient anti-tau antibody for this purpose (Additional file 2: Fig. 2). IP from either 1 or 5 ml of CSF vs. PBS recovered equivalent tau seeding activity (Fig. 4a) indicating that neither the volume of the IP, nor matrix effects of CSF proteins reduced seed recovery. Next we spiked 1 ml aliquots of control CSF with successive dilutions of protein from AD frontal cortex or recombinant tau fibrils and performed immunopurification followed by the seeding assay. Seeding from the IP eluent was compared with seeding from direct treatment of biosensor cells with the corresponding quantity of AD brain protein or synthetic fibrils (Pre-IP). We detected seeding activity in CSF from as little as 31.6 pg of total AD brain protein (Fig. 4b) and 100 attomoles (monomer equivalent) of recombinant fibrils (Fig. 4c). The enhanced sensitivity with IP for AD-derived tau but not for synthetic tau fibrils is likely due to use of the TauA antibody, which may not bind as efficiently to heparin-treated synthetic fibrils that have a distinct structure [11, 44]. Similarly, IP of AD brain protein enhances seeding over that of the equivalent pre-IP brain homogenate (Fig. 4b) which is not seen with fibrils (Fig. 4c). This reflects an inhibitory effect of brain homogenate on seeding that we see consistently in our experiments, and highlights the importance of IP from biological samples prior to seeding.

### Absence of tau seeding activity from pre-mortem AD CSF

**Fig. 5:**
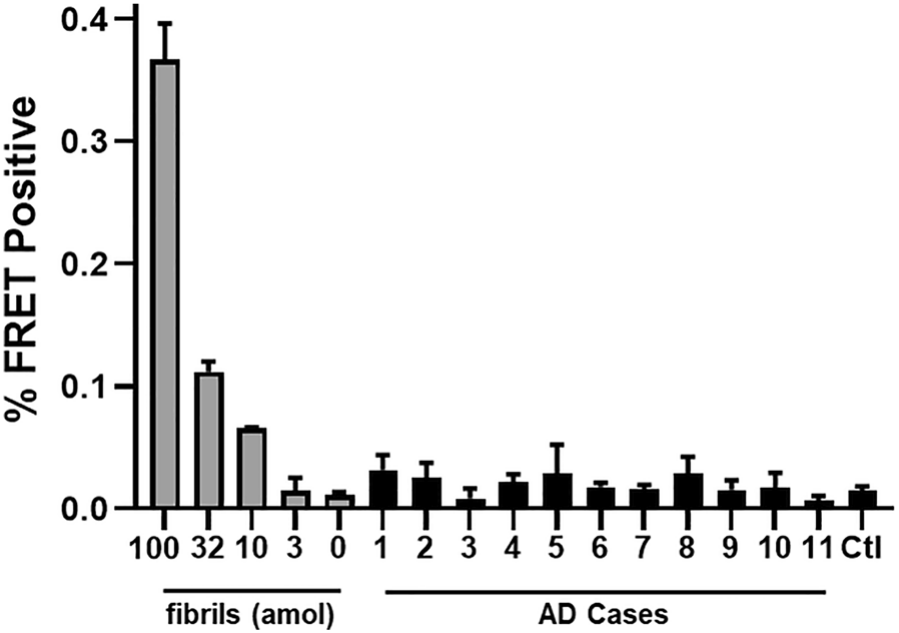
Absent CSF seeding in v2H biosensor cells. Results are from immunopurification from 1 ml CSF. The left end of the graph shows FRET positivity from a serial dilution of synthetic tau fibrils. * LLD = 10 attomoles of tau. Error bars are SEM of 3 technical replicates over which each sample was divided

To test for seeds in CSF, we obtained 1 ml CSF samples from 11 subjects with a diagnosis of probable AD. In 9 subjects the diagnosis of AD was supported by validated CSF biomarkers [39]: elevated CSF p-tau and decreased Aβ_42_ (Table 1). We performed IP using the TauA antibody, followed by the seeding assay as in Fig. 4. A standard curve of recombinant tau fibrils established a lower limit of detection of 10 attomoles (monomer equivalent) for the seeding assay. Nonetheless, we detected no seeding activity above baseline from any of the CSF samples. We conclude that within the limits of the most sensitive biosensor system available we cannot detect tau seeds in the presence of highly probable AD (Fig. 5).

**Table 1:** CSF biomarker values for the 11 AD patients tested

Patient	Age	Sex	CSF t-tau (pg/ml)	CSF p-tau (pg/ml)	CSF Aβ_42_ (pg/ml)	ATI
UT-1	69	F	993.4	99	303.15	0.21
UT-2	76	M	398	75.95	395.25	0.56
UT-3	69	F	1768.8	193.25	226.9	0.10
UT-4	71	M	869.85	128.9	296.1	0.23
UT-5	68	M	NA	NA	NA	NA
UT-6	51	M	NA	NA	NA	NA
UT-7	63	M	1137.45	132.9	373.55	0.24
UT-8	63	M	1098	128.65	233.2	0.15
UT-9	76	F	430.3	73.6	279.85	0.37
UT-10	91	M	538.3	85.4	446.4	0.51
UT-11	74	F	562.65	98.25	255.85	0.28
NC	64	F	134.95	32.15	561.45	1.41

CSF is considered consistent with AD if p-tau > 68 pg/ml and ATI < 0.8. ATI is defined as Aβ_42_/(240 + 1.18*t-tau). NC is normal control

## Discussion

Tau assemblies that act as templates for their own amplification (seeds) may underlie progression of neurodegenerative tauopathies, and assays that measure the levels of these pathogenic forms thus have great utility. While highly sensitive and specific conformational antibodies would be ideal, amplification of tau seeds in purified systems [27, 29–31] or in cultured “biosensor” cells has been the mainstay of sensitive and specific detection of pathological tau. Biosensor cells created by our group or others are now widely reported to detect pathological forms of tau [4, 7, 10, 13, 15, 16, 21, 24, 25, 34, 35, 38, 42]. Reliable measurement of tau seeding activity in a peripheral fluid such as CSF could be very useful in detection of incipient tauopathy. Consequently, we have tried repeatedly and unsuccessfully to detect pathological tau in human CSF or blood. We reasoned that a more sensitive biosensor system might solve the problem. We optimized expression of tau RD(P301S)-Clo/Cer in HEK293T cells and created a biosensor with 100-fold improved sensitivity versus the original line [21]. Interestingly, this increase was noted only with AD-derived seeds and not with synthetic heparin-treated fibrils (Fig. 2). This is unlikely to be due to differences in average fibril size as we have found no differences in the curves with synthetic fibrils sonicated for five minutes to decrease the fibril size (Additional file 3: Fig. 3). The reason for this difference remains to be determined, but it likely involves a preference of the P301S RD construct for the structure of the AD strain over those of synthetic fibrils, which are known to have distinct tertiary structure [11, 44]. The v2H line should be especially useful to quantify tau seeds that are of low abundance. Given their linear dynamic range, the v2L line may be more useful to quantify seeding in samples with stronger signal. The utility of the original v1 biosensor assays to detect early evidence of tau pathology in brain tissue has already been demonstrated, and we anticipate that the v2H cell line will enhance detection of pathological tau beyond current capabilities.

Considering the high sensitivity of the v2H cells to detect pathological tau, we were surprised at our failure to detect seeding activity in CSF from antemortem AD subjects, as this has been reported previously by others [31, 36, 37]. We can envision several reasons for this. First, there may be differences in the way samples were prepared and added to biosensor cells. Alternatively, though we have never observed cross-reactivity between tau and other amyloid proteins in the biosensor cells, other factors present in CSF samples could trigger tau aggregation, rendering false positive results.

Another possibility is that published RT-QuIC assays of tau seeding exceed the cell-based biosensor system in sensitivity. RT-QuIC assays have demonstrated tau seeding in post-mortem CSF, though with less sensitivity than in brain [30, 31]. Post-mortem CSF may contain intracellular tau released after death, and thus pre-mortem CSF is a more accurate reflection of clinical utility. A 4R RT-QuIC assay sensitive for PSP and CBD seeds showed higher mean signal in groups of PSP and CBD pre-mortem CSF relative to a group of controls but not with sufficient sensitivity and specificity to apply clinically [31]. Sensitivity of the RT-QuIC assays is demonstrated by consistent amplification from extremely dilute samples of brain homogenate. By combining IP with the v2H seeding assay we detected seeding from 31.6 pg of total brain protein diluted in 1 ml of CSF, representing a dilution factor of about 10^9^. This is very similar to the published sensitivities of 3R and 4R tau RT-QuIC assays for seeding from AD, Pick, PSP, and CBD brain homogenates [29–31].

Finally, it is possible that seed-competent conformations of tau are confined to the brain and do not enter the CSF. To have seeding activity, tau species must contain portions of the RD, which forms the core of all known tau fibrils [9, 11]. Quantification of tau sequences by MS indicates that the proline-rich mid-region (aa197-243) of tau is most abundant in human CSF while sequences within the RD are much rarer [2, 3]. Similar results have been obtained using IP-MS [8], and plate-based immunoassays with antibody pairs targeting different regions [12]. This pattern of fragments differs considerably from that found in the brain. Stable isotope labeling kinetic studies of tau metabolism and turnover in human neurons have found a regulated truncation and secretion of tau species containing only N-terminal and mid-regions [33], which may explain the observed pattern of CSF tau fragments. While total tau levels in the CSF can rise due to passive release with neuronal death, such as in acute stroke [19], elevated CSF tau in AD patients represents truncated, rather than full-length species [3], indicating that it is likely driven by differences in processing and secretion. Seed-competent tau can be released into the extracellular fluid in cell culture models [26, 41], but so far, except for one study [37], it has been difficult to document seeding activity in the interstitial fluid, let alone the CSF.

While RD fragments probably constitute a minority of tau species in human CSF, several studies have detected appreciable concentrations, reporting levels in the low pg/ml [2, 3, 8, 33]. Notably, Blennow et al*.* recently published a novel Simoa assay of tau fragments ending at amino acid 368 (tau368), the C-terminus of RD. They found concentrations of tau368 in CSF from AD patients averaging about 20 pg/ml [5]. Due to the highly fragmented nature of CSF tau, previous MS-based studies may have been limited by immunocapture with antibodies against N-terminal or mid-region epitopes, or with low affinity for RD fragments. Recently, Horie et al. have used an antibody-independent method of solid-phase protein extraction paired with MS to measure ng/ml concentrations of tryptic tau RD fragments in CSF. Importantly, these tryptic fragments do not reflect the native fragmentation pattern in CSF, and while three RD fragments were elevated in AD CSF relative to control, they differed in their concentration in CSF and correlation with disease stage. This suggests that the RD fragments in CSF may differ from seed-competent RD in the brain and might not contain the intact AD amyloid core region.

If even a tiny fraction of RD-containing tau species leaks from the brain into the CSF, the ultrasensitive assay should detect seeding in AD. Horie et al*.* measured tau RD fragments in the CSF at 400 pg/ml. Thus it is interesting that The v2H biosensor detects seeding from as little as 31.6 pg of total protein from an AD brain, of which 0.89% is tau as measured by ELISA. This corresponds to a concentration of 281 fg/ml of AD-derived tau in the spiked CSF sample. Therefore we would expect to detect seeding if the RD tau fragments in AD CSF were at all representative of tau in the brain. The lack of detection suggests a mechanism acting specifically on seed-competent tau to sequester it in the brain. Seed-competent conformations of tau RD may be less likely to enter the CSF due to their propensity to aggregate, analogous to the decrease in CSF Aβ42 in AD [22], or due to a binding affinity for HSPGs on the surface of cells [20]. This may limit the usefulness of tau seeding as a biomarker. Regardless, the ability of the v2H cell line to quantify trace amounts of tau seed from biological specimens may prove useful in further studies of tauopathy.

## Supplementary Information

**Additional file 1.** Cell lines v2L and v2H have complementary linear dose-response ranges. Plots of percent FRET positivity show non-linear kinetics for v2L between 316 fg and 316 pg (A), but a high degree of linearity between 316 pg and 100 ng (C) (r^2^ = 0.997). Conversely, v2H has a highly linear dose response between 316 fg and 316 pg (B) (r^2^ = 0.998), but is non-linear between 316 pg and 100 ng 21 (D).

**Additional file 2.** TauA antibody efficiently purifies seeds from dilute AD brain homogenate. Seeding activity was measured in the IP and supernatant fractions of IPs of 50 ng of AD brain protein with the TauA rabbit polyclonal antibody against a.a. 244-266 and a mouse monoclonal antibody against a.a. 22-34 (equivalent to HJ8.5).

**Additional file 3.** Sonication of fibrils does not alter the dose-response curves on biosensor cell lines. We prepared synthetic fibrils in the standard way, which includes a brief 30 second water bath sonication prior to dilution (A), and with 5 minutes of water bath sonication to decrease the average length of the fibrils (B). The relative curves were not different.

## Declarations

### Availability of data and materials

Data sets from flow cytometry experiments are banked in the CytoBank digital repository and links to datasets supporting individual figures will be provided by the corresponding author upon request. TauRD P301S v2L and v2H biosensor cells will be submitted to ATCC and until then will be made available upon request, as will the TauA antibody.
