# Supplementary figures and images for: Ultrasensitive tau biosensor cells detect no seeding in Alzheimer’s disease CSF
Source: Acta Neuropathol Commun. 2021 May 26;9:99. doi: 10.1186/s40478-021-01185-8 (PMC8152020; doi:10.1186/s40478-021-01185-8)

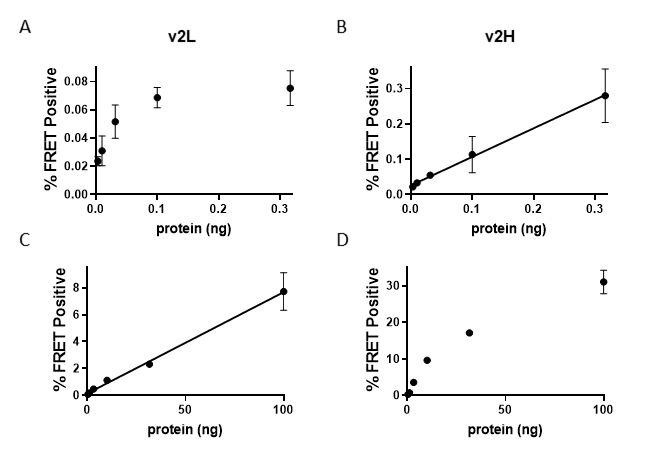

Supplement: Supplementary file 1 — Additional file 1. Cell lines v2L and v2H have complementary linear dose-response ranges. Plots of percent FRET positivity show non-linear kinetics for v2L between 316 fg and 316 pg (A), but a high degree of linearity between 316 pg and 100 ng (C) (r2 = 0.997). Conversely, v2H has a highly linear dose response between 316 fg and 316 pg (B) (r2 = 0.998), but is non-linear between 316 pg and 100 ng 21 (D). [file 40478_2021_1185_MOESM1_ESM.tif]

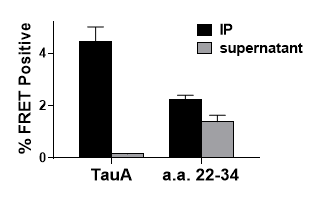

Supplement: Supplementary file 2 — Additional file 2. TauA antibody efficiently purifies seeds from dilute AD brain homogenate. Seeding activity was measured in the IP and supernatant fractions of IPs of 50 ng of AD brain protein with the TauA rabbit polyclonal antibody against a.a. 244-266 and a mouse monoclonal antibody against a.a. 22-34 (equivalent to HJ8.5). [file 40478_2021_1185_MOESM2_ESM.tif]

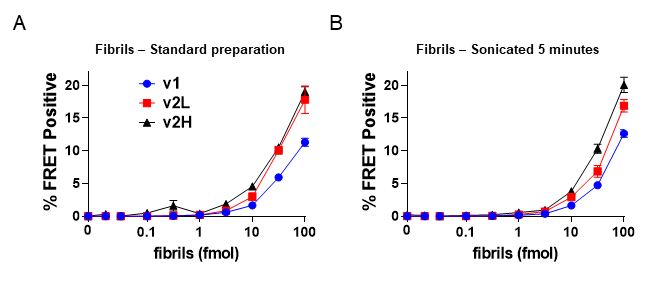

Supplement: Supplementary file 3 — Additional file 3. Sonication of fibrils does not alter the dose-response curves on biosensor cell lines. We prepared synthetic fibrils in the standard way, which includes a brief 30 second water bath sonication prior to dilution (A), and with 5 minutes of water bath sonication to decrease the average length of the fibrils (B). The relative curves were not different. [file 40478_2021_1185_MOESM3_ESM.tif]
